# The Ketogenic Diet: Breath Acetone Sensing Technology

**DOI:** 10.3390/bios11010026

**Published:** 2021-01-19

**Authors:** Omar Alkedeh, Ronny Priefer

**Affiliations:** Department of Pharmaceutical Sciences, Massachusetts College of Pharmacy and Health Sciences University, Boston, MA 02115, USA; oalke1@stu.mcphs.edu

**Keywords:** breathalyzer, acetone, ketogenic diet, semi-conductor metal oxides

## Abstract

The ketogenic diet, while originally thought to treat epilepsy in children, is now used for weight loss due to increasing evidence indicating that fat is burned more rapidly when there is a low carbohydrate intake. This low carbohydrate intake can lead to elevated ketone levels in the blood and breath. Breath and blood ketones can be measured to gauge the level of ketosis and allow for adjustment of the diet to meet the user’s needs. Blood ketone levels have been historically used, but now breath acetone sensors are becoming more common due to less invasiveness and convenience. New technologies are being researched in the area of acetone sensors to capitalize on the rising popularity of the diet. Current breath acetone sensors come in the form of handheld breathalyzer devices. Technologies in development mostly consist of semiconductor metal oxides in different physio-chemical formations. These current devices and future technologies are investigated here with regard to utility and efficacy. Technologies currently in development do not have extensive testing of the selectivity of the sensors including the many compounds present in human breath. While some sensors have undergone human testing, the sample sizes are very small, and the testing was not extensive. Data regarding current devices is lacking and more research needs to be done to effectively evaluate current devices if they are to have a place as medical devices. Future technologies are very promising but are still in early development stages.

## 1. Introduction

A ketogenic diet has many variations in micronutrients, timing, portion size, frequency of meals, and caloric restriction, but the main tenet is reduced carbohydrate intake. A precursor to the modern ketogenic diet was first introduced for epileptic children in 1911 where physicians in France used starvation as a method of controlling seizures and noted positive progress, although without quantifiable evidence [1]. Subsequent studies have shown that there may be some benefit, specifically with a low carbohydrate diet in controlling children’s epilepsy [2,3]. At the Mayo Clinic, RM Wilder coined the term “ketogenic diet” after he observed the increased ketone levels in the patients that had reduced carbohydrate intake [4]. The modern ketogenic diet has now become a popular method of weight loss, and has lost favor in seizure control, due to more advanced antiepileptic medications. Many people struggle with obesity because of the prevalence of highly carbohydrate dense meals, including ingredients such as high fructose corn syrup. The ketogenic diet has been shown to aid with weight loss when combined with exercise [5,6,7]. With this popularity, the ketogenic diet, including supplements, foods, education, and devices has a market of around $10 billion and a projection of $15 billion by 2027 [8].

**Figure 1:**
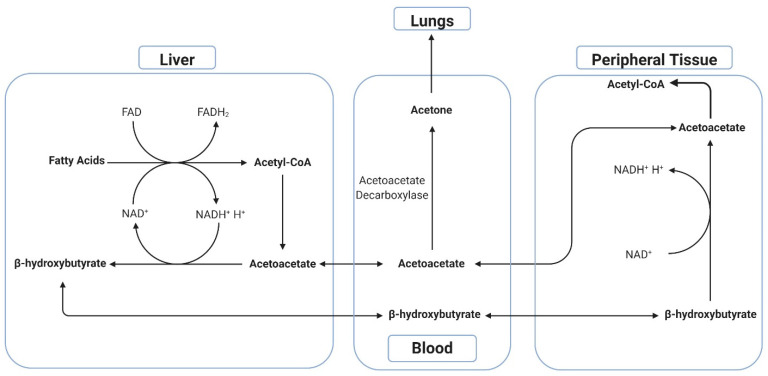
Human ketone metabolic pathways summary.

The human breath contains hundreds of volatile organic compounds (VOCs) [9,10] as first noted by Linus Pauling in 1971 [11]. The most recognizable of these markers is ethanol through the use of alcohol breathalyzers [12]. While ethanol in the breath dictates the same compound in the blood, acetone is not present in the blood in a significant quantity but is rather a metabolic step in the breakdown of fatty acids. This breakdown yields three compounds of interest with respect to the ketogenic diet: specifically, β-hydroxybutyrate (BHB), acetoacetate (AcAc), and acetone [13]. These ketone bodies are markers of a ketogenic state in which the body is not using glucose for energy, but rather free fatty acids. The term “ketogenic diet” or “ketodiet” is a derivation of ketone-genesis, or the making of ketones. This state of ketosis is hypothesized to assist in weight loss by increasing the rate of lipolysis [5,6,7,14,15]. The breaking down of fatty acids leads to AcAc production in the blood, which is then further metabolized into BHB and acetone. The majority of acetone is formed when AcAc is enzymatically decarboxylated via acetoacetate decarboxylase located in various tissues throughout the body (Figure 1). This process is dependent on blood glucose levels, with a higher level of glucose correlating with lower levels of acetone production, while lower levels of glucose correlate with higher acetone production [13]. In states of starvation, or low glucose, the body needs energy in the form of ATP and this lipolysis is essential in supplying the ketone bodies needed to produce it. It is hypothesized that by measuring the levels of either AcAc, BHB, or acetone, an estimated level of ketosis can be gauged [2,5,7,14,16,17,18,19,20,21,22,23].

Acetone specifically can be measured in the breath and has been shown to correlate with the level of ketosis such as BHB detection in the blood [2,17]. Other VOCs have been explored as biomarkers for the diagnosis and management of disease states and monitoring of lifestyle. These can range from epilepsy [2,3], various cancers [24,25], diabetes [17,26], lactose intolerance [27] exposure to pollutants [9], and ketogenic diets where the level of acetone measured in exhaled air has been reported to correlate with the degree of ketosis [2,3,5,6,7,14,18,28,29]. This is of interest to those on a ketogenic diet because it indicates “how well” their diet is working and if adjustments are needed. It is important for dieters to know where they are along the axis to help guide their decisions and for type 1 diabetics to avoid ketoacidosis, a severe ketogenic state characterized by extremely high ketone levels and very low blood glucose which could result in a coma [30].

This zone of ketogenesis that has been suggested to be optimal is in the range of 1.5–3.0 mmol/L of BHB, if measuring blood ketones, and 2–40 ppm of acetone if measuring in the breath [14]. Falling within this range would inform dieters if they are in a state of ketosis. Breath acetone (BrAce) and BHB have been positively correlated showing R^2^ values ranging from 0.7 [18] to 0.9 [2,5]. Blood BHB is routinely measured in clinics across the country as a normal laboratory assay. BrAce has only recently come into popularity due to emerging technologies. Early renditions of acetone breathalyzers used similar redox reactions as their ethanol counterparts in the hopes of crossover, but ultimately lacked specificity. Modern renditions of the breathalyzers have adopted newer technologies to increase sensitivity and specificity. Many current approaches employ the use of semiconductor metal oxides as a reactive sensor [31,32,33,34,35]. These can take many forms, with respect to the metal used and physicochemical arrangements. A small portion of BrAce meters on the market utilize photoionization detectors which detect acetone’s absorbance under different wavelengths to determine the concentration present in the breath [36]. Future technologies currently in development mostly revolve around development of novel metal oxides to increase specificity, sensitivity, and durability.

## 2. Current Technology

Current ketone sensing technologies employ mass spectrometry, photoionization, gas chromatography, light-addressable potentiometric sensors, quantum cascade lasers, and semiconductor metal oxides [26]. These approaches can be used to detect ketones in the urine, breath, and blood. Previous technologies predominately involved the use of mass spectrometry and gas chromatography. However, these technologies are limited due to their cost and bulk. Tests would need to be ordered with the patient’s samples being sent to the lab which would take time to receive results. Because of the cost and inconvenience, these tests were not routinely done. The current standard of care for diabetics is to check glycated hemoglobin and blood glucose levels using a blood test to mostly monitor high levels of glucose. Ketone sensing technology has been proposed as a supplemental diagnostic tool for high levels of ketones [26].

Current trends in ketone sensing technologies revolve around handheld breath acetone detectors. These devices are capable of detecting levels of acetone in the breath usually in ppm levels and report it to the user. Utility of this with respect to the ketogenic diet comes from linking the degree of ketosis to BrAce. Some key factors need to be identified before confidently accepting the breathalyzer’s readout. These include the patient health, the device has high sensitivity and specificity for acetone, the dieter has not ingested anything that could interfere with the signal, and the timing between meals and exercise. Additionally, there are some identified factors that affect BrAce readout, some of which are; exercise, obesity, garlic ingestion, disulfiram, and variability in body temperatures [7]. Exercise has been shown to increase BrAce in adults because energy in the form of glucose has been depleted and the body moves to metabolize fat stores [37]. Obese individuals have been shown to have lower levels of BrAce. This mechanism is not fully understood and may be related to insulin resistance where fat oxidation is limited [21]. Garlic ingestion has been shown to moderately increase BrAce by possibly inhibiting acetone metabolism [38]. Disulfiram, a drug used to treat alcohol dependence, may also increase BrAce through its acetaldehyde dehydrogenase inhibition. Temperature extremes also have effects on overall metabolic rate in humans and variations may correlate with BrAce changes [39]. These factors are important to consider when using single point measurements of BrAce. Repeated measurements over a day provide ketone exposure rather than immediate ketosis status which may be more useful for long term dieters.

The majority of current devices use semiconductor metal oxide (SMO) sensors to detect BrAce. This is due to the cheap manufacturing cost, portability, low energy input, and the wide range of evidence supporting their accuracy [26,40,41,42,43]. Many SMOs are available with sensitivity and specificity to acetone. A number of current commercially available devices are generic versions with no patent that have been rebranded by multiple companies [35]. This makes it difficult to determine their exact makeup, technology, and reliability.

Evidence for currently available commercial ketone breathalyzers is lacking. Many studies have assessed the utility of novel sensors in the ketogenic diet [25,41,42,43,44,45,46,47,48,49,50,51,52,53,54,55,56,57,58,59,60,61,62,63,64,65,66,67,68], but very few have evaluated currently available devices. The BIOSENSE™ device is a breathalyzer with published clinical trial data detailing its efficacy. However, the data reported in this trial should be taken lightly because an independent reviewer was not present and the sample size was very small. The parent company, Readout Health, has published prospective observational cohort study of 21 subjects using the BIOSENSE™ and Precision Xtra devices. The Precision Xtra is an FDA cleared device from Abbot Laboratories, Inc., Abbot Park, IL, USA that measures blood ketones and glucose [69]. The subjects recruited were majority female (81%), described as ethnically diverse, and divided between a ketogenic diet and standard diet. They self-reported daily values for blood ketones and BrAce. Values were measured five times throughout the day and subjects were adherent at a rate of 100%, 93%, and 63% to three, four, and fives daily tests, respectively. A total of 1214 measurements comparing BrAce and blood ketones were run in a linear regression analysis and obtain the coefficient of determination to be R^2^ = 0.57 (*p* < 0.0001) [70]. Additionally, measurements from each individual were plotted separately for each day giving a total of 248 total subject days and used to examine the daily ketone exposure (DKE). The value derived from the area under the curve (AUC) of ketone concentration over time [70]. This DKE value for both BrAce and blood BHB was analyzed and another linear regression was calculated giving R^2^ = 0.80 [70].

This Readout Health study produced valuable results with respect to DKE. The authors demonstrated that ketone levels throughout the day are highly variable, thus limiting the utility of single point measurements. By measuring multiple times throughout the day, a DKE calculation can be obtained to give overall ketone exposure. More data is needed to determine the utility of DKE calculations in comparison to single point readings. The BIOSENSE™ device measures single point readings, but a mobile application also determines a DKE calculation called a “Ketone Score” [70].

However, the utility of the BIOSENSE™ device cannot be accurately assessed. There are many limitations to the study and not enough data. The sample size of the device is only 21 which is very low for a clinical trial. The subjects’ baseline characteristics were never described in the study and only mentioned to be ethnically diverse which limits the generalizability of the results. There was a lack of gender distribution with 80% of the subjects being female. The DKE calculation was determined using days that had four or five readings and those readings had adherences of 93% and 63%, respectively which again limits the sample size and generalizability. The specific build of the device was only mentioned as a metal oxide semiconductor with no details beyond that. The subjects’ diet was also not monitored even though the authors recognized that certain factors and drugs can influence BrAce. This study was also conducted by the same company that produced the BIOSENSE™ device with no data handling by a third party, so accuracy of the data is questionable.

Another reported device is the METRON disposable acetone sensor. This device is not currently commercially available but has a mechanism unique to other acetone sensors. The device is described by US Patent 8,871,521 [71] as a hollow tube with a liquid and powder component. The subject would break a seal and combine the two components and then blow through the device. A reaction between sodium nitroprusside (SNP) and an ammonium salt would occur facilitated by acetone in the breath. The level of BrAce would correlate with reaction extent via colorimetric spectroscopy. A purple color would be observed with 1.4 mg/dL of BrAce, or a “positive” result. This device is no longer commercially available.

The INVOY breathalyzer contains a liquid nanoparticle sensor as detailed in US Patent 9,486,169 [72]. The specific metal oxide was not mentioned, and no studies have evaluated its effectiveness. It is described in the patent as containing liquid cartridges that can react with BrAce and produce a colorimetric change that is detected by a camera and relayed to a sensing system which then gives an approximate BrAce level.

The Ketoscan device developed by Sentech GMI corp. in Korea uses a photoionization detector calibrated for acetone, as described in its patent application [73]. There is no published data describing its specificity or sensitivity or use in a ketogenic diet. The Ketoscan company has posts on its website claiming of undergoing clinical trials; however, there are no published results in any scientific journal and only simple results comparing the detected fat lost with the device to actual fat lost are indicated; however, this data is not currently verifiable.

Other ketone breathalyzers listed in Table 1 have very limited data on their functionality, build, and effectiveness. Some can be assumed to be SMO containing due to similar user steps and build, but no data is currently available evaluating these devices. Their accuracy with respect to specificity and sensitivity is questionable and may or may not be provided. More studies are needed to evaluate current available ketone sensing systems. FDA status is also indicated in the table because it may indicate the intentions of the manufacturer and quality of the device.

End-tidal breath or the last air out of a breath is noted to contain the true concentration of acetone [74]. What this means for sensors is that a sustained breath is needed to accurately detect the breath acetone level. Currently available sensors do not indicate they have a specific mechanism for this, but most direct the user to exhale for around 10 s which gets around the end-tidal volume of air for healthy people.

## 3. Future Technologies

The main sensors presented here are metal oxides and light-based sensors. Semiconductor metal oxides at their basic level detect resistance changes along their surface when exposed to the different gases. Oxygen is present on their surface which is reduced, and that electron changes the resistance of the sensor. These sensors are no longer plain metal oxides but consist of many different nano-arrangements and can also be doped with other metals, changing their porosity and overall function. The challenge many researchers have is to find the right ratio of metals to combine, an adequate nano-arrangement, and a cheap and simple process for development [80]. Light based sensors are also presented as an alternative to the bulk gas chromatography and mass spectrometry. They emit light that is then picked up by a sensor and in between the emitter and sensor is the gas. Certain gases affect the light differently and this can be calibrated into the sensors to find the exact gas concentration present [43].

### 3.1. Metal Oxides and Organic Based Sensors

Many sensors in development are utilizing metal oxides as its base and experimenting with augmentations to the metal in an effort to increase the sensitivity, specificity, efficiency, and durability. Plain metal oxides have some utility in sensors, but do not have the sensitivity and selectivity of the novel augmented sensors being researched today. Other metals in certain nano conformations, such as nanosheets, nanoparticles, and nanotubes are being researched to allow for more VOC and oxygen adsorption to their surface and increase selectivity toward acetone. One variation between the sensors seen is the operating temperature. This is the temperature that the researchers have selected for the device to operate at in order to achieve maximum response. This temperature is not sustained but exists for a small amount of time in the sensor. A lower operating temperature would be ideal because a lower power consumption and higher temperature tend to lower stability of the sensors. Here we explore some BrAce sensors utilizing metal oxides and discuss their effectiveness and utility. A summary of highlighted data is provided on Table 2.

#### 3.1.1. Zinc Oxide, Cadmium Based

Graphene quantum dots (GQD) are nanostructures with use in many different applications including gas sensing. Liu et al. [54] have developed a GQD-functionalized three-dimensional ordered mesoporous (3DOM) ZnO sensor for acetone detection. The goal was to improve upon basic ZnO sensors and demonstrate a manufacturing process for assembling the sensor. First polymethyl methacrylate (PMMA) spheres were permeated with ZnO(NO_3_)_2_ and underwent a calcination process to remove volatiles, yielding a 3DOM ZnO structure. GQDs were made by dispersing graphite powder with strong acids. This solution was subsequently washed, dried, and heated to produce the GQDs. The 3DOM ZnO were spin-coated with the GQDs to produce the sensor backbone and tested on various gases. Operating temperature of this sensor was lower than other 3DOM ZnO sensors (320 °C vs. 380 °C) which is crucial since there is a concern of decomposition of the GQDs at temperatures greater than 350 °C. The addition of the GQD yielded faster response and recovery times (at 1 ppm of acetone GQD 3DOM ZnO (9/16 s) vs. 3DOM ZnO (16/27 s). The incorporation of the GQD sensor also led to a lower detection limit of 50 ppb vs. 200 ppb with the 3DOM ZnO sensor. With regards to selectivity, the GQD 3DOM ZnO sensor exhibited poor response to water and was highly selective to acetone compared to other gases (R_air_/R_gas_ = 15.2 at 1 ppm). NO_2_, H_2_S, NH_3_, toluene, ethanol, methanol, isopropanol, and NO were tested with values lower than 4.1. As for stability, the sensor was measured to have lost 3% weight when subjected to temperatures from 50–200 °C. This weight loss was attributed to the decomposition of water present in the samples by the authors. The authors described that this sensor has potential utility as a diabetic diagnostic tool to detect high ketones in the breath, but the same concept can be applied to a ketogenic diet [54].

Researchers at Shenzhen University in China have developed two ZnO based sensors which are Au or Pd doped to enhance the acetone sensing capabilities [53]. The sensor was prepared using microwave assisted solvothermal reactions. The Au doped sensors achieved a maximum response of R_air_/R_gas_ = 102 at 150 °C with 2.0 wt% Au, while the Pd doped sensor achieved a maximum response of R_air_/R_gas_ = 69 at 150 °C with 1.5 wt% Pd. Selectivity for acetone was compared to ethanol, methanol, H_2_, NH_3_, and SO_2_. Both sensors displayed selectivity, with the Au doped sensor being more selective to acetone. The response and recovery times of the Au doped sensor was 8 and 5 s, respectively, while the plain ZnO sensor was 9 and 7 s, respectively. Sensor stability was noted to be excellent by the authors with only slight variation in response at 150 °C [53].

Šetka et al. developed a new sensor capable of working at room temperature using cadmium telluride/polypyrrole (CdTe/PPy) nanocomposites which were integrated into Love mode surface acoustic wave (L SAW) sensors [50]. This new sensor achieved high specificity and selectivity for VOCs, including acetone. The sensor was made up of two components, the first being PPy, which is a conductive polymer, and the second being CdTe. The PPy was meant to overcome weaknesses associated with plain conductive polymers such as high working temperature, poor selectivity, or low response speed [50]. By incorporating a metal in the form of CdTe, these limitations could be overcome. The CdTe quantum dots (QD) were first formed using CdCl_2_, tri-sodium citrate dihydrate, and mercaptopropionic acid (MPA). The pH was adjusted, and sodium tellurite and sodium borohydride were added. The solution was mixed and heated to form the CdTe QDs which were combined with the polypyyrole by mixing the solutions and then spin coating. CdTe and PPY were combined in two different concentrations (CdTe/PPy 1:10 and 1:2) and another sensor was left with only PPy. The 1:10 CdTe/PPy sensor showed the highest response while the 1:2 CdTe/PPy sensor showed the lowest. The PPy sensor had sensitivity to acetone (652 Hz/ppm), while the 1:10 CdTe/PPy sensor was improved with approximately 1.2× greater sensitivity. Overall, this technique and new sensor is promising; however, human testing needs to be conducted to assess its function once exposed to the hundreds of VOCs found within the human breath. A positive attribute to this sensor is the ability to operate at room temperature with low energy requirement.

Researchers in Vietnam have synthesized ultrathin porous ZnO nanoplates via a urea mediated synthesis [66]. These nanoplates are 2D structures which are hypothesized to have excellent sensitivity due to their exposed planes. The nanoplates were synthesized via a hydrothermal method. A scanning electron microscope was used to observe the structures and demonstrate their ultrathin and porous structure which is thought to contribute to their gas sensing ability. The sensor was tested with acetone, ethanol, methanol, toluene, and NH_3_ gases at temperatures ranging from 350 to 450 °C. The sensor was found to be temperature dependent with a decrease in temperature leading to decreased response. At 125 ppm of acetone, the sensor had a response of 20 (R_air_/R_gas_). The response and recovery time for the sensor were calculated from transient resistance versus time when flow was switched from air to gas and back to air. The sensor had response and recover times at acetone concentration of 50 ppm, of 23 and 637 s, respectively and at concentrations of 50–125 ppm, of 7 and 550 s, respectively. Compared to the aforementioned GQD 3DOM ZnO [53] and the CdTe/PPy [50] sensors, the response/recovery time is very high. The operating temperature of 450 °C is also very high and would require a greater energy input. Despite the high temperature, the sensor displayed excellent stability over 10 exposures to acetone over 10,000 s with little variation in response.

A miniaturized gas chromatographic column utilizing ZnO QD was developed by researchers in the Republic of Korea [43]. These QDs were synthesized using a: Zn acetate, *N*,*N*-dimethylformamide, and tetramethylammonium hydroxide then laid on the sensor. A patient would breathe into a sampling loop, and a pump would slowly push 1 mL of the sample through a packed column. The acetone was then detected on the ZnO QDs based on changes in the resistance of the sensor. Using this method, the gasses are physically separated before to ensure greater accuracy. Responses were recorded in terms of (Δlog(R)) and were positively correlated with stock acetone gas in ppm with an R^2^ of 0.9915. The device was tested on three subjects with one undergoing a ketogenic diet. It was noted there was an increased response correlating with higher BrAce in the subject undergoing the ketogenic diet. The correlation is very strong at R^2^ = 0.9915 [43]; however, the sample size of 3 is very small with only a few data points, and thus not enough to draw conclusions about the device’s utility.

#### 3.1.2. Iron Oxides

La_x_FeO_3_ based sensors have rising popularity with researchers looking to optimize and enhance current sensors. La_x_FeO_3_ has stability at high temperatures and can be formed into specific crystal arrangements to allow ultralow detection limits of acetone. Chen et al. in China have developed nano-LaFeO_3_ thick-films in an attempt to reach ppb levels of acetone detection [41]. To make their sensor, lanthanum nitrate, ferric nitrate, and citric acid were mixed and formulated using the sol-gel method, then heated in an oven and mixed with water to make a paste to spread onto the sensor plate. The sensor was determined to have the highest response when annealed at 800 °C. The sensor exhibited different responses depending on the concentration of acetone. For acetone concentrations of 0.5 ppm, 1 ppm, 5 ppm, 10 ppm, the responses were 2.068, 3.245, 5.195, and 7.925 (R_air_/R_gas_), respectively. Ethanol and acetone were compared at 5 ppm and ethanol had a higher response, 2.44 vs. 1.936 showing poor selectivity. Compared to other gases such as HCHO, NH_3_, and methanol, the sensor had greater selectivity for acetone. Optimal operating temperature was determined to be 260 °C with response times from 37–51 s and recovery times of 82–155 s.

A Bi_1−x_La_x_FeO_3_ (x = 0–0.2) sensor was developed by Peng at al. in China [47]. The authors’ goal was ultralow levels of acetone detection. A sol-gel method was used to make the sensor using: Bi(NO_3_)_3_·5H_2_O, Fe(NO_3_)_3_·9H_2_O, and La(NO_3_)_3_·6H_2_O mixed in acids and then heated to yield the final powder, which was cast on a gold sensor plate. Using Bi_1−x_La_x_FeO_3_ (x = 0.1) at 50 ppb acetonitrile, tetrahydrofuran, N-hexane, CHCl_3_, CH_2_Cl_2_, NH_3_, and xylene were tested alongside acetone and there was no detectable response, while with acetone there was a response of 8 (R_air_/R_gas_). The highest response achieved was 40 (R_air_/R_gas_) at 100 ppm of acetone and 260 °C. The sensor was tested in 55%, 70%, and 90% RH with response decreasing as the RH was increased and acetone test gas concentration decreased. The lowest response was 2.8 (R_air_/R_gas_) at 90% RH and 50 pbb of acetone and the highest was around 16 (R_air_/R_gas_) at 55% RH and 1000 ppb. Response/recovery time was exceptional at 260 °C and 15 and 13 s, respectively. The sensors were tested for stability over 4 weeks and found little variation in response when tested at 50, 600, and 1000 ppb of acetone. This sensor was not tested on human subjects, but has been proposed as a diabetic or ketogenic diet device [47].

#### 3.1.3. Tin Oxides

Li et al. in China incorporated Pd and Au onto SnO_2_ nanosheets (NSs) for another sensor. This sensor was made to compare the effects of Pd and Au on enhancing the gas sensing performance of SnO_2_ nanosheets. To make the sensor, a hydro-solvothermal method was used to produce SnO_2_ nanosheets. Pd and Au were then added along with ascorbic acid to produce the doped nanosheets. Two separate sensors with only either Pd or Au on the SnO_2_ nanosheets were also made for comparison. The Pd/Au SnO_2_ sensor outperformed bother sensors in all categories and required a lower operating temperature. The sensor was not tested on humans, but the authors noted that under 94% RH the sensor performed well. It exhibited a 45 ppb detection limit which is very low compared to other sensors and had a response of 6.5 (R_air_/R_gas_) at 2 ppm of acetone under an operating temperature of 250 °C and very fast response/recovery times of 5/4 s possibly owing the fast times to a highly exposed surface area and porosity from the unique nanosheet structure. This sensor was made as an enhancement of a basic SnO_2_ sensor to demonstrate how Pd and Au can improve the sensor. Compared to a basic SnO_2_ sensor, the PdAu/SnO_2_ sensor has over three times the response to acetone at 1 ppm. Stability over 40 days was tested and a response reduction of 3–5% was detected when measuring at 250 °C [48].

Similarly, another SnO_2_ sensor was developed by Cho et al. [63] to maximize gas sensing performance by increasing porosity. This was done by using SnO_2_ spheres made via a novel electrostatic spraying method which also incorporated Pt into the nanostructures resulting in Pt-pore-loaded hierarchical SnO_2_ (Pt-PH-SnO_2_) spheres. These were compared to PH-SnO_2_ spheres and hierarchical SnO_2_ spheres. The benefit of the new Pt-PH-SnO_2_ spheres as compared to previous sensors is the pores which allow for about a 20% enhanced gas sensing response. This sensor also demonstrated a novel electrostatic spraying technique which allows an easier and more robust method of SMO sensor synthesis. The maximum response of the Pt-PH-SnO_2_ sensor was 44.83 (R_air_/R_gas_) at 5 ppm compared to a much lower 6.61 (R_air_/R_gas_) from the PH-SnO_2_ sensor. The operating temperature is on the higher end at 400 °C. The detection limit is 200 ppb which is intermediate compared to other sensors discussed. The response time of the Pt-PH-SnO_2_ sensor was noted to be 7 s, but no acetone concentration was given [63]. The recovery time was noted to be fast; however, no value was mentioned. This sensor was evaluated in 90% RH and exhibited similar performance with or without humidity. Stability of the sensor was noted to be great. Measurements were taken at 18 months and the response was reduced to 34.18 at 5 ppm. No human tests were conducted and more data on this sensor is needed, such as complete response and recovery times and accuracy compared to other sensors.

#### 3.1.4. Tungsten Oxides

Tungsten Oxides are very popular in current BrAce sensor research. Ding et al. have developed a WO_3_ sensor and compared its functionality when doped with chromium [52]. This sensor follows the process of Cr doping from research in 2008 to form a similar sensor [82]. The sensors were not tested on humans but was tested in 40% RH. The sensor had a low detection limit of 298 ppb, an operating temperature of 250 °C, and a response of 71.52 (R_air_/R_gas_) at 100 ppm. The plain WO_3_ sensor was noted to have a response three times lower than the chromium doped one. Through thorough investigation by Ding et al., tungsten annealed at 450 °C and 100 mg of chromium were determined to give the best response [52]. Tungsten oxide was also used in another sensor made and tested by Kim et al. in Korea and the United States. WO_3_ nanofibers incorporated with Pt nanoparticles were encapsulated in a protein cage and then heat treated to give the desired dispersion of Pt nanoparticles. The sensor was tested under varying RH up to 90%, but not tested on humans. The response/recovery times and detection limits were not addressed by the researchers. A response of 88.04 (R_air_/R_gas_) at 5 ppm was noted. This sensor utilizes a novel method of a sacrificial template in the form of a protein which allows for a different method of dispersing nanoparticles on a medium [42].

Platinum and palladium have been historically used in their pure form as electrodes for various electronic application. Current uses, with respect to breath acetone sensors, involve the addition of Pt or Pd in the sensor to enhance its functionality. One such sensor has been developed by Deng et al. in China where WO_3_·H_2_O hollow spheres were modified with 0.02% PtCu nanocrystals to improve response by up to 9.5 times [56]. Deng at al.’s sensor exhibited maximum response of 204.9 (R_air_/R_gas_) with 0.02% PtCu at a temperature of 280 °C using a stock 50 ppm acetone sample [56]. Their sensor also demonstrated good selectivity for acetone when compared to other gases, including the following: methylbenzene, ethanol, formaldehyde, methanol, benzene, ammonia, and hydrogen with acetone achieving a response 4 times greater than any of the aforementioned gases, but was not tested for in humid conditions nor tested on humans [56]. An early tungsten-based sensor from 2012 was developed by researchers from Switzerland and Austria using Si-doped WO_3_ nanostructured films [49]. This sensor has limited data on its capabilities such as maximum response. The fast response and recovery times of 10 and 35 s, respectively, and very low detection limit of 20 ppb seem very promising. Revisiting this sensor may be warranted to possibly optimize it and further test with humans. It was tested on five healthy male participants in 2012 with limited data regarding the tests. It seemed to exhibit very high selectivity when comparing the acetone response to the second best response of ethanol with a value of around 18. This may not accurately represent the sensor as a whole due to only testing against ethanol in the 2012 study. This sensor was further tested in 2013 on 8 healthy subjects to correlated glucose levels and breath components and found a strong correlation of 0.98 after overnight fasting [82]. Further testing of this sensor on 20 volunteers in 2017 on fat metabolism showed that the elevated breath acetone post exercise was correlated with elevated blood BHB and that the sensor is suitable as an exercise monitor [83]. A year later in 2018, this sensor was tested again with a ketogenic diet and 11 volunteers and found that a ketogenic diet raised ketone levels. The breath acetone level was compared to blood BHB and the levels were in agreement. The authors noted that the sensor could be suitable for tracking a ketogenic diet along with ketosis status of an epileptic patient. Further research on this sensor incorporated it into a sensing array for human detection in collapsed buildings. The array would include this Si:WO_3_ sensor, a Si:MoO_3_ sensor, and a Ti:ZnO sensor. This array was set to detect acetone, ammonia, and isoprene concentrations at 19, 21, and 3 ppb respectively. This was a unique approach to using breath acetone monitoring [84]. Lastly in 2020, the sensor was enhanced with a catalytic filter made of Al_2_O_3_ nanoparticles which was heated to 135 °C before the sensor was heated to 400 °C. This allows it to have an enhanced selectivity of over 250 against many analytes including alcohols, aldehydes, aromatics, isoprene, ammonia, hydrogen gas, and carbon monoxide. Stability was excellent with responses staying constant when subjected to 145 days at 90% relative humidity [81].

#### 3.1.5. Nickel Oxides

Nickel containing sensors have some popularity in gas sensors and a current research exists to try and find optimal chemical and physical configurations. Liu et al. in China have developed a nickel-based sensor using stabilized zirconia and NiTa_2_O_6_. Their sensor had a detection range of 0.2–200 ppm of acetone, sensitivity of −11/−27 (mV/decade), response and recovery times in seconds of 9 and 18, respectively. The selectivity was not great with ethanol and methanol achieving around half of the response to acetone under various conditions. The response of the sensor fluctuated between −2.5–1.5 mV in relative humidity ranges of 20–98%, which demonstrates good applicability for human breath. The operating temperature of 600 °C was quite high; however, the sensor demonstrated good stability over repeated use [61]. Another nickel-based sensor was developed by researchers from India and Italy using MgNi_2_O_3_ nanoparticles. The sensor did not have a linear response for detecting acetone concentration, but a calibration was possible to determine a concentration based on previous known values. The sensor has an operating temperature of 200 °C, which is much lower compared to the aforementioned NiTa_6_O_6_ sensor. The response to acetone at 10 ppm was 2.3 (R_air_/R_gas_). A detection limit was extrapolated to be 0.3 ppm with values only measuring down to 0.5 ppm. The response and recovery times were 25 and 250 s, respectively. The sensor was also proposed to be able to directly measure blood glucose levels. The long-term stability of the sensor was not discussed; however, the sensor was noted to be stable over the fluctuations of the test with a constant response. This sensor was not tested in humid conditions nor tested on human subjects [59].

### 3.2. Light Based

New sensors are under development utilizing light absorbance and diffraction. Lasers are often used for these applications due to their precision and stability which allows for more accurate measurements [85,86,87,88,89]. Schwarm et al. have developed a laser sensor that has been tested on subjects undergoing a ketogenic diet [37]. The sensor follows classic Beer–Lambert law by measuring absorbance of a gas and comparing it to known levels of acetone to determine the concentration present in exhaled breath. The sensor has an array of fiber optic cables and mirrors to lay out the laser path. This sensor uses an 8.2 µm region which was determined to give the least interference. Accuracy has only been tested against known concentrations of acetone in N_2_ gas in a testing chamber and not compared with gas chromatography or mass spectroscopy in humans. Human testing was done to determine if the sensor can detect changes in BrAce but not for accuracy. Five subjects were enrolled out of UCLA medical center and underwent a ketogenic diet. They had baseline BrAce measured before the diet and began a 36-h ketogenic diet. All subjects demonstrated elevated BrAce from baseline for the duration of the diet and 3 h post diet. The sensor was the exclusive BrAce measuring device used and accuracy of the results are not clear. This sensor is in the early stages and seems promising if more human testing is done.

Another light-based sensor from the UK uses direct Ultraviolet (UV) absorbance to measure acetone [45]. Acetone of known concentrations were measured using the sensor and gas chromatography. The sensor was tested on 10 healthy human subjects against gas chromatography and showed a linear correlation with an R = 0.971 [45]. The human breath samples were collected into Tedlar^®^ bags and not directly measured with either gas chromatography or the sensor to obtain more consistent results. This sensor also had a detection limit of 0.7 ppm. Being a light-based sensor, the values governing the sensors are different than the SMOs and a direct comparison may be difficult. The largest advantage of this sensor is the use of cheap UV LEDs which also have a low energy requirement compared to traditionally employed lasers.

### 3.3. Comparisons

Certain metal oxides are used because of their cheap cost and are easy to work with such as zinc and iron, which can be acquired cheaply. Tungsten is sought after as a sensor because of its unique ability to be formed in complex crystalline and nanoparticle morphologies. Current research does not include bare metal oxides and most sensors are doped with other metals such as Cr, Pd, Pt, Au, and Si in hopes of enhancing their abilities. The versatility of these metal oxides comes at a price, however. Historically, these bare metal oxides had poor stability with repeated exposure to humidity and the various analytes. Progress has been made to enhance stability, but there is a limit to this because the sensor will always be in direct contact with the analytes and humidity. Light based sensors can be beneficial because of this, and allow for a closed loop system to protect the sensors and offer long term stability such as Li et al.’s [45].

The more prominent sensors discussed here are the semiconductor metal oxides. They differ in their material, operating temperature, output data (selectivity, sensitivity, response), and the extensiveness of their testing. Humidity is necessary to test in because human breath is very humid and before human testing can begin, the sensor must be validated in humid conditions. Many sensors test in humid conditions and even test long term stability in humid conditions which is very valuable such as Weber et al.’s sensor [82]. More than half the sensors discussed here were not tested on humans. In the early stages of development this is acceptable, but to further pursue them as commercial devices, they should undergo the testing. The selectivity of the sensors is very important to differentiate between different VOCs. The highest selectivity (250) was seen with Weber et al.’s sensor [81]. This is due to the catalytic process added to eliminate other analytes before they reach the sensor. The maximum response was also noted for each sensor and the highest was noted with Deng et al.’s sensor with a response of 204 (R_air_/R_gas_) at 50 ppm. The maximum response is important because it needs to be significant enough to be detected, but also it needs to vary from other analytes to have good selectivity. The response and recovery times are also crucial because it allows for convenience and ease of use. A sensor with a long response or recovery time might not see commercial success. The lowest times reported were with Li et al.’s sensor 5/4 s response and recovery times respectively at 2 ppm of acetone. The operating temperature is another crucial parameter. A high operating temperature can be dangerous and thus requires significant power to use the sensor. A lower temperature is preferred to also keep the sensor stable.

For a manufacturer to decide on the use of the different technologies, it comes down to their needs. A high operating temperature sensor may be suitable if it is cheap and the company can afford the excess power needed in a new power delivery system. A sensor with low selectivity may find success with a company that then adds a step before it to reduce other analytes such as Weber et al.’s design with the catalytic step prior [81]. There is not a sensor that is best all around, but there are certainly sensors that have valuable traits.

## 4. Conclusions

The rising popularity of the ketogenic diet is expanding the use of acetone breathalyzers owing to, in part, the data demonstrating the possible benefit of the diet with respect to diabetes management and weight loss. This popularity has founded a new market for acetone breathalyzers. Many breathalyzers are currently on the market, but there is a lack of scientific evaluation of these sensors. Most sensors do not have any data regarding their utility for use in a ketogenic diet. Novel sensors being developed provide some of the data and testing, but more is still needed. Novel sensors have been developed utilizing SMOs, lasers, UV light, nanoparticles, and organic scaffolds. SMO sensors typically containing basic metal oxides such as zinc, iron, and tungsten, however, are being phased out in favor of sensors doped with other metals such as gold, platinum, and palladium and/or nanoparticles. There is a significant body of research on these sensors regarding their sensitivity, selectivity, efficiency, and durability. Many of these novel technologies report high responses to acetone; however, many of these have not undergone human testing and may show conflicting results compared to simulated breath in the lab. Selectivity is especially an area of concern because the sensors need to differentiate between the different analytes. Some sensors have not reported selectivity data and others have only reported against a few analytes which does not adequately simulate real world applications. To more adequately compare the various sensors on the market and in development, more rigorous testing is needed which can pave the way for more FDA registered devices.

## Figures and Tables

**Table 1:** Current commercially available breath acetone sensing devices.

Brand	Technology	FDA Status	Strengths	Limits
METRON [71]	SNP ^c^, ammonium sulfate powder	N/A ^b^	Disposable	Off Market
INVOY [72,75]	Liquid Cartridges with Metal Oxide ^d^	Registered Class 1	Available through a nutrition program	Disposable cartridges
Ketoscan [36,73]	Photoionization Detector	Registered Class 1	Undergoing trials	Limited data
House of Keto [35]	Metal Oxide Detector	N/A ^b^	Cheap cost	Generic build, Limited data
Ketonix [76]	N/A ^a^	Registered Class 1	Cheap cost	Limited data
Qetoe [34]	Metal Oxide Detector	N/A ^b^	Cheap cost	Generic build, Limited data
Lencool [33]	Metal Oxide Detector	N/A ^b^	Cheap cost	Generic build, Limited data
Lexico health [32]	Metal Oxide Detector	N/A ^b^	Cheap cost	Generic build, Limited data
KetoPRX [77]	N/A ^a^	N/A ^b^	Cheap cost	Generic build, Limited data
Keyto [78]	N/A ^a^	N/A ^b^	Easy use	Limited data
LEVL [79]	N/A ^a^	Registered Class 1	Clinician coaching included	Expensive, limited data
Biosense [31,70]	Metal Oxide Detector	Registered Class 1	Data available	expensive

^a^ Technology in use was not publicly available. ^b^ The device listed is not registered with the FDA or the status could not be determined. ^c^ Sodium Nitroprusside (SNP). ^d^ Specific details regarding composition of liquid and the metal oxide was not listed.

**Table 2:** All discussed semiconductor metal oxide (SMOs) and organic based sensors.

Technology	Operating Temp (°C)	Detection Limit (ppb)	Response/Recovery Time (s)	Maximum Response (R_air_/R_gas_) ^a^	Reference	Selectivity (Max Response/2nd Best Response)	Relative Humidity Tested	Tested on Human Breath? (Y/N)
MgNi_2_O_3_	200	500	25/250 (40 ppm)	2.3 (10 ppm)	Lavanya et al. [59]	~1.87	N/A	N
NiTa_2_O_6_	600	200	9/18 (2 ppm)	N/A	Liu et al. [61]	~1.5	20–98%	Y
PtCu/WO_3_·H_2_O HS	280	10	3.4/7.5 (50 ppm)	204.9 (50 ppm)	Deng et al. [56]	~5.4	N/A	N
PdAu/SnO_2_	250	45	5/4 (2 ppm)	6.5 (2 ppm)	Li at al. [48]	~2.1	40–70%	N
Cr/WO_3_	250	298	N/A	71.52 (100 ppm)	Ding et al. [52]	~4.3	25–90%	N
Apo-Pt@HP WO_3_NFs	350	N/A	N/A	88.04 (5 ppm)	Kim et al. [42]	~2.95	90%	Y
ZnO QDs	430	100	N/A	N/A	Jung et al. [43]	N/A	N/A	Y
3DOM ZnO	320	8.7	9/16 (1 ppm)	15.2 (1 ppm)	Liu et al. [54]	~3.75	25–90%	Y
Au (2%)/ZnO nanorod	150	5	8/5 (100 ppm)	102 (100 ppm)	Huang et al. [53]	~3.4	N/A	N
Pd (1.5%)/ZnO nanorod	150	5	9/7 (100 ppm)	69 (100 ppm)	Huang et al. [53]	~2.6	N/A	N
CdTe/PPY	24	5	155/270–310 (5 ppm)	N/A	Šetka et al. [50]	N/A	30%	N
Bi_0_._9_La_0_._1_FeO_3_	260	50	15/13 (50 ppb)	40 (100 ppm)	Peng et al. [47]	5.71	55–90%	N
ZnO nanoplates	450	45	23/637 (50 ppm)	20 (125 ppm)	Van Duy et al. [66]	2.22	10–80%	N
Pt-PH-SO_2_	400	200	7/(N/A)	44.83 (5 ppm)	Cho et al. [63]	~3.57	90%	N
Si:WO_3_	350	20	14/36 (100 ppb)	N/A	Righettoni [49]	18	0–90%	Y
Catalytic enhanced Si:WO_3_	400	50	55/100 (500 ppb)	4.3 (1 ppm)	Weber et al. [81]	250	90%	Y

^a^ Maximum response recorded without humidity or other interfering gases.
